# A Vehicle Routing Optimization Problem for Cold Chain Logistics Considering Customer Satisfaction and Carbon Emissions

**DOI:** 10.3390/ijerph16040576

**Published:** 2019-02-16

**Authors:** Gaoyuan Qin, Fengming Tao, Lixia Li

**Affiliations:** College of Mechanical Engineering, Chongqing University, Chongqing 400044, China; qingaoyuan@cqu.edu.cn (G.Q.); lilixia@cqu.edu.cn (L.L.)

**Keywords:** vehicle routing problem, cold chain logistics, customer satisfaction, carbon emissions, carbon trading

## Abstract

Under fierce market competition and the demand for low-carbon economy, cold chain logistics companies have to pay attention to customer satisfaction and carbon emissions for better development. In order to simultaneously consider cost, customer satisfaction, and carbon emissions in the cold chain logistics path optimization problem, based on the idea of cost–benefit, this paper proposes a comprehensive cold chain vehicle routing problem optimization model with the objective function of minimizing the cost of unit satisfied customer. For customer satisfaction, this paper uses the punctuality of delivery as the evaluation standard. For carbon emissions, this paper introduces the carbon trading mechanism to calculate carbon emissions costs. An actual case data is used with a cycle evolutionary genetic algorithm to carry out computational experiments in the model. First, the effectiveness of the algorithm and model were verified by a numerical comparison experiment. The optimization results of the model show that increasing the total cost by a small amount can greatly improve average customer satisfaction, thereby obtaining a highly cost-effective solution. Second, the impact of carbon price on total costs, carbon emissions, and average customer satisfaction have also been numerically analyzed. The experimental results show that as carbon price increases, there are two opposite trends in total costs, depending on whether carbon quota is sufficient. Increasing carbon price within a certain range can effectively reduce carbon emissions, but at the same time it will reduce average customer satisfaction to a certain extent; there is a trade-off between carbon emissions and customer satisfaction. This model enriches the optimization research of cold chain logistics distribution, and the study results complement the impact research of carbon price on carbon emissions and customer satisfaction. Finally, some practical managerial implications for enterprises and government are offered.

## 1. Introduction

With the continuous improvement of people’s living standards and the increasing demand for fresh food, the cold chain logistics industry has developed rapidly. In order to effectively reduce the cost of cold chain logistics distribution, some scholars have also begun to study the optimization of cold chain logistics distribution networks [1,2]. In fact, optimizing cold chain logistics distribution networks not only considers the general cost of items, but also considers customer satisfaction and carbon emissions.

Customer satisfaction is a customer’s feelings about products and services received [3]. In many cases, satisfaction is known to be of great value in understanding customers’ perceptions and evaluations [4]. Creating and retaining satisfied customers is essential for the success of businesses [5]. Researchers have pointed out that the higher customer satisfaction is, the higher customer loyalty, intention of repetitive purchasing, positive word-of-mouth, and market share will be [6,7,8]. Therefore, good customer satisfaction can bring potential value to a business. For logistics companies, cold chain logistics distribution is a highly competitive service industry [9]. As one of the determinants of service quality, responsiveness stands as a main driver for differentiation, and this is evaluated by promptness of the service delivery [10]. Therefore, it is becoming more and more important that goods can be delivered within the time ranges desired by customers in this era of fierce market competition. As such, it is wise to pay attention to customer satisfaction when planning cold distribution routes.

In addition, rapid economic growth is accompanied by high consumption, resulting in large amounts of carbon emissions [11], so reducing greenhouse gas emissions has become a hot issue in the world [12]. At the Copenhagen Climate Summit, China made a commitment that by 2020, carbon intensity would be reduced to 40–45% lower than in 2005 [13]. Global carbon emissions data show that the transportation industry produces 14% of the total carbon emissions, while road transportation produces more than 70% of the entire transportation industry’s emissions [14]. Cold chain logistics is a particularly high energy and high carbon emissions industry [15]. Therefore, how to reasonably arrange cold distribution paths to reduce energy consumption and carbon emissions is a serious problem we have to face. The carbon trading mechanism is one of the most effective measures of controlling carbon emissions [16,17,18]. Carbon dioxide emission rights are used as a commodity, thus forming a carbon dioxide emission right transaction, referred to as carbon trading.

In summary, we must not only consider customer satisfaction but also environmental benefits in cold distribution process. Therefore, we must consider these questions: How to evaluate customer satisfaction in cold chain logistics distribution; how to quantify carbon emissions; and how to build a comprehensive optimization model that considers cost, customer satisfaction, and carbon emissions. Thus, this paper is organized as follows: A literature review of related work is presented in Section 2. The model formulation is proposed in Section 3. A cycle evolutionary genetic algorithm is described in Section 4. The algorithm experiment and case experiment are shown in Section 5. Discussion and managerial implications are illustrated in Section 6. Finally, conclusions are presented in Section 7.

## 2. Literature Review

The main idea of this paper is to obtain an optimal distribution plan with consideration of customer satisfaction and carbon emissions in cold chain logistics distribution. We review the literature about VRP (vehicle routing problem) in three areas: The research about cold chain logistics, the research about customer satisfaction, and the research about carbon emissions.

### 2.1. Research about Cold Chain Logistics

There is a series of research about VRP model in cold chain logistics. Hsu et al. [19] studied a VRP with time windows for perishable food delivery, and constructed a SVRPTW (stochastic vehicle routing problem with time windows) model by considering the randomness of the perishable food delivery process. Then, Amorim et al. [20] also conducted a study on the distribution of perishable goods through a case; they studied a heterogeneous fleet site-dependent vehicle routing problem with multiple time windows and used an adaptive large neighborhood search algorithm to solve the problem. In order to ensure delivery of safe, fresh, high quality foods to customers, Hsiao et al. [21] introduced quality level into cold chain logistics, and an algorithm based on adapting biogeography-based optimization (BBO) was developed. Ji et al. [22] studied VRP with simultaneous delivery and pickup for cold chain logistics. Liu et al. [23] studied the optimization of cold chain logistics distribution network terminals, and found that the semi-trailer is used to carry out the transportation of jammers by using mathematical modeling and time window constraint analysis, which can effectively integrate cold chain logistics resources, save logistics space and human resources, and improve terminal logistics distribution capacity. Bao and Zhang [24] studied the route optimization of cold chain logistics in joint distribution, and declared that joint distribution outperforms partition distribution not only in distribution costs but also in carbon costs.

From the research on cold chain distribution above, we see that these studies are aimed at achieving the lowest cost, with no attention to customer satisfaction or environmental factors.

### 2.2. Research Considering Customer Satisfaction

In the field of VRP research, some scholars have conducted research on customer satisfaction. Since customer satisfaction is an abstract concept, scholars have also proposed different calculation methods. Cheng and Gen [25,26] introduced the fuzzy due-time, and used triangle fuzzy numbers to describe customer satisfaction. Zhang et al. [27] proposed an improved fuzzy due-time window to express customer satisfaction. Fan [28] claimed that the shorter the waiting time, the higher the satisfaction, and established a multi-objective function to minimize the total transportation cost and maximize the overall customer satisfaction. Guerriero [29] proposed a method of event soft windows to calculate satisfaction. Ghannadpour et al. [30] used a function of fuzzy time windows to express satisfaction when studying multi-objective dynamic vehicle routing problem. Bakeshloo et al. [31] also adopted function of fuzzy time windows to measure customer satisfaction, and proved that there are trade-offs between customers satisfaction, total costs, and emissions.

In summary, although scholars have different expression functions about customer satisfaction, the same parts exist in those functions: When the arrival time is within the time window that meets the requirement of the customer, they will be completely satisfied. Furthermore, since cold chain logistics focuses on the distribution of perishable or fresh products, customers put forward more stringent requirements for timely delivery to ensure freshness [32]. Therefore, for the problem of cold chain distribution, this paper assumes that if the vehicle arrives within the time window requested by the customer, the customer will be completely satisfied; if not, they will be dissatisfied. This paper also defines the average customer satisfaction as the proportion of fully satisfied customers to the total number of customers.

### 2.3. Research Considering Carbon Emissions

Recently, many scholars have begun to pay attention to environmental issues, and a large number of VRP models considering carbon emissions have emerged. Wang et al. [15] studied the impact of carbon tax on carbon emissions in the cold chain distribution process. Then, Wang et al. [33] also studied the impact of carbon tax on carbon emissions in an inventory routing problem. Shen et al. [34] studied the impact of the carbon trading mechanism on carbon emissions in multi-depot open VRP. Niu et al. [35] proposed a green open VRP with time windows by minimizing comprehensive routing cost, which included fuel cost, carbon emissions cost, and driver cost. Liao et al. [36] proposed a hybrid meta-heuristic algorithm to solve the on-line VRP for minimizing costs related to economics and emissions. Guo and Liu [37] proposed a cost function including driver cost and fuel cost in the time-dependent pollution-routing problem. Naderipour et al. [38] proposed a new comprehensive model for the measurement, evaluation, and minimization of CO_2_, NO_x_, and CO as three important emissions in the open time-dependent vehicle routing problem.

In summary, there is still little research on cold chain transportation that considers the three factors of economy, customer satisfaction, and environment at the same time. Thus, this paper proposes a comprehensive VRP-CSC model (vehicle routing problem for cold chain logistics considering customer satisfaction and carbon emissions) to optimize distribution paths. In order to minimize the total costs, including the carbon emissions costs, and maximize customer satisfaction, based on the idea of cost–benefit [39,40], this model adopts the ratio of cost to customer satisfaction as the objective function, instead of minimization of the total costs. Detailed design processes of the objective function in this paper are shown in Section 3.2. Because China is setting up a national carbon trading market, the carbon trading mechanism was introduced into this model to calculate carbon trading costs.

**Figure 1:**
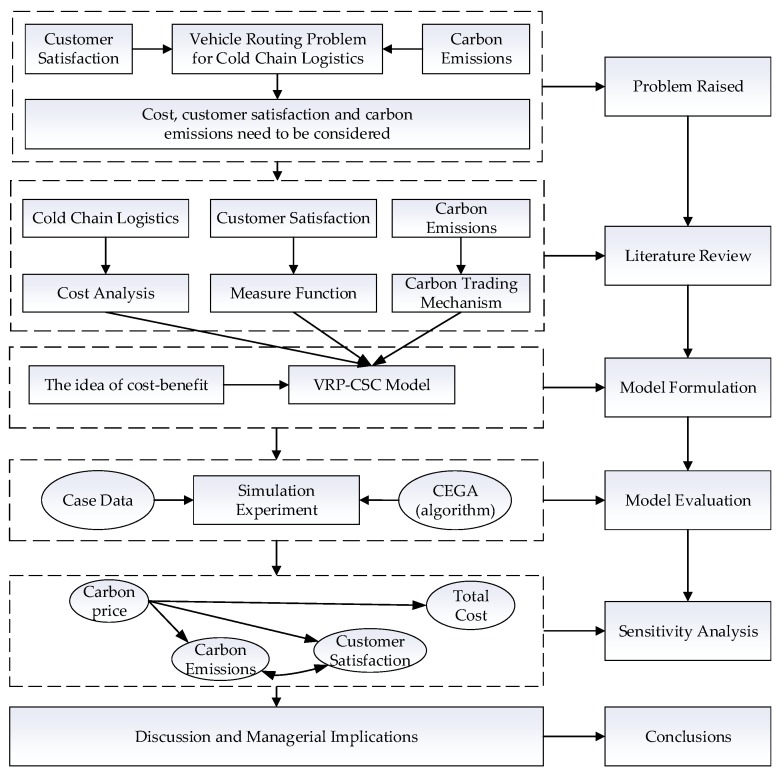
Flowchart of the study.

Due to the premature maturity of standard genetic algorithms, the cycle evolutionary genetic algorithm (CEGA) is used to solve the model in the paper. The algorithm is proposed based on catastrophism theory, simulates the phenomenon of evolution and degradation coexisting in the evolution of nature, and shows the characteristic of cyclical reciprocation [41]. At the same time, a combination operator [42] is designed to ensure the algorithm can obtain the optimal solution. This operator can guarantee the continuous evolution of the population and presents as a spiral rise in general. The flowchart of this paper is shown in Figure 1.

## 3. Model Formulation

### 3.1. Problem Description

There is a refrigerated central depot with a certain number of transport vehicles, and a set of customers need to be served. The locations of the depot and each customer are known, and the demands of each customer are known. In the meantime, all trucks must return to the depot when their distribution tasks are completed. In addition, different customers have different demands for time window. If the vehicle arrives within the time window requested by the customer, the customer will be completely satisfied; otherwise, they will be dissatisfied. Additionally, we also need to consider the issue of carbon emissions. In short, the main purpose is to find an optimal solution considering the factors of cost, customer satisfaction, and environment. The detailed assumptions in this paper are as follows:

(1) There is only one type of truck, and the total load on each route cannot exceed the rated load of the vehicle.
(2) The locations of the depot and each customer are known, and the demand of each customer is known.
(3) All trucks must return to the depot when their distribution tasks are completed and each customer is only visited once.
(4) If the vehicle arrives within the time window requested by the customer, the customer will be completely satisfied; otherwise, they will be dissatisfied.

### 3.2. Objective Function of the VRP-CSC

All the research on customer satisfaction in the literature review is to establish the following multi-objective optimization model:

(1) $$\{\begin{array}{l} min\sum operational\;costs\;\;\;\;\;\;\;\;\;\\ max\sum customer\;satisfaction \end{array}$$

The result obtained according to this model is a Pareto solution set. However, decision makers are still unable to make good judgments about how to choose a better plan from these non-inferior solutions. Therefore, based on the idea of cost–benefit, this model adopts the ratio of cost to customer satisfaction as the objective function, instead of minimization of the total costs. Thus, this paper establishes a highly cost-effective objective function as follows:

(2) $$min\frac{\sum operational\;costs}{ACS}$$

Equation (2) ensures that the cost of unit average customer satisfaction is minimized. The average customer satisfaction (ACS) of this paper is the proportion of fully satisfied customers to the total number of customers.

(3) $$ACS=\frac{\sum_{i=0}^{N}\theta_{i}}{N}$$

Because the total number of customers N is a fixed value in a delivery task, the final objective function in this paper can be expressed as:

(4) $$min\frac{\sum operational\;costs}{\sum_{i=0}^{N}\theta_{i}}$$

This objective function ensures that the cost of unit satisfied customer is minimized. The solution of the objective function is equivalent to selecting the most cost-effective solution from the Pareto solution set derived from the multi-objective function.

### 3.3. Symbols

Based on the needs of building the model, this paper uses the corresponding symbols which are listed in Table 1.

### 3.4. Sub-Costs of Model

(1) The fixed cost of vehicles

When a truck is called, some fixed costs need to be paid, including the cost of the driver’s wages, truck wear and tear, road maintenance fees, and so on. Thus, the fixed costs$\;C_{1}\;$can be expressed as:

(5) $$C_{1}=\overset{K}{\underset{k=1}{\sum }}\overset{N}{\underset{i=1}{\sum }}\left(Y_{0ik}f_{k}\right)$$

(2) The damage costs

Reference [15] pointed out that the damage costs consist of the damage costs$\;C_{21}\;$for travel and the damage costs$\;C_{22}\;$for the open door process. The damage costs$\;C_{21}\;$for travel can be expressed as:

(6) $$C_{21}=\overset{N}{\underset{i=0}{\sum }}\overset{N}{\underset{j=0}{\sum }}\overset{K}{\underset{k=1}{\sum }}Z_{i}pq_{i}\left(1-e^{-\partial_{1}\left(t_{i}-t_{0}\right)}\right)$$

When the door is opened, due to the convection of air, the temperature inside the vehicle will rise, and the spoilage rate will also rise, so the damage cost$\;C_{22\;}\;$for the open door process can be expressed as:

(7) $$C_{22}=\overset{N}{\underset{i=0}{\sum }}\overset{N}{\underset{j=0}{\sum }}\overset{K}{\underset{k=1}{\sum }}Z_{i}pQ_{in}\left(1-e^{-\partial_{2}s_{i}}\right)$$

Thus, the total damage costs$\;C_{2}\;$can be expressed as:

(8) $$C_{2}=\overset{N}{\underset{i=0}{\sum }}\overset{N}{\underset{j=0}{\sum }}\overset{K}{\underset{k=1}{\sum }}Z_{i}p\left[q_{i}\left(1-e^{-\partial_{1}\left(t_{i}-t_{0}\right)}\right)+Q_{in}\left(1-e^{-\partial_{2}s_{i}}\right)\right]$$

(3) The refrigeration costs

As for damage costs, reference [15] pointed out that the refrigeration costs consist of the refrigeration costs$\;C_{31\;}$in travel and the refrigeration costs$\;C_{32}\;$for the open door process.

(9) $$C_{31}=C_{e}\overset{N}{\underset{i=0}{\sum }}\overset{N}{\underset{j=0}{\sum }}\overset{K}{\underset{k=1}{\sum }}X_{ijk}\left(t_{j}-t_{i}\right)$$

(10) $$C_{32}=C_{e}{}^{\prime }\overset{N}{\underset{i=0}{\sum }}\overset{N}{\underset{j=0}{\sum }}\overset{K}{\underset{k=1}{\sum }}X_{ijk}s_{j}$$

Thus, the total refrigeration costs$\;C_{3}\;$can be expressed as:

(11) $$C_{3}=\overset{N}{\underset{i=0}{\sum }}\overset{N}{\underset{j=0}{\sum }}\overset{K}{\underset{k=1}{\sum }}X_{ijk}\left[C_{e}\left(t_{j}-t_{i}\right)+C_{e}{}^{\prime }s_{j}\right]$$

(4) The fuel consumption costs

Some scholars have come up with a linear function for fuel consumption [43]. The linear function formula of fuel consumption per unit distance is as follows:

(12) $$\rho \left(X\right)=\rho_{0}+\frac{\rho^{*}-\rho_{0}}{Q}X$$

Therefore, the total fuel consumption in the entire distribution can be expressed as:

(13) $$F_{c}=\overset{N}{\underset{i=0}{\sum }}\overset{N}{\underset{j=0}{\sum }}\overset{K}{\underset{k=1}{\sum }}\rho \left(Q_{ij}\right)d_{ij}$$

Thus, the total fuel consumption costs$\;C_{4}\;$in the distribution process can be expressed as:

(14) $$C_{4}=\overset{N}{\underset{i=0}{\sum }}\overset{N}{\underset{j=0}{\sum }}\overset{K}{\underset{k=1}{\sum }}X_{ijk}C_{r}\left(\rho_{0}+\frac{\rho^{*}-\rho_{0}}{Q}Q_{ij}\right)d_{ij}$$

(5) The carbon emissions costs

Kwon [44] first introduced the carbon trading mechanism to the VRP. Li et al. [45] further studied the impact of the carbon trading mechanism on logistics distribution. These studies point out that when the actual carbon emissions are lower than the carbon quota allocated, companies can sell carbon emission rights to gain profit, and if the carbon emissions are greater than the upper limit, the company must purchase additional carbon subsidies. Thus, the carbon trading costs$\;C_{5\;}$in distribution process can be expressed as:

(15) $$C_{5}=C_{p}\left(\emptyset F_{c}-Q_{q}\right)$$

### 3.5. Setting of VRP-CSC

Through the comprehensive analysis above, the VRP-CSC model is given by the following:

(16) $$\min r=\frac{C_{1}+C_{2}+C_{3}+C_{4}+C_{5}}{\sum_{i=0}^{N}\theta_{i}}$$

Subject to:

(17) $$\overset{K}{\underset{k=1}{\sum }}\overset{N}{\underset{i=1}{\sum }}Y_{0ik}\leq K\;$$

(18) $$\overset{N}{\underset{i=1}{\sum }}Y_{0ik}=\overset{N}{\underset{i=1}{\sum }}Y_{i0k}\leq 1,\;k=1,2,\ldots ,K$$

(19) $$\overset{K}{\underset{k=1}{\sum }}\overset{N}{\underset{i=0}{\sum }}X_{ijk}=1,\;j=1,2,\ldots ,N$$

(20) $$\overset{K}{\underset{k=1}{\sum }}\overset{N}{\underset{j=0}{\sum }}X_{ijk}=1,\;i=1,2,\ldots ,N$$

(21) $$\overset{N}{\underset{i=0}{\sum }}\overset{N}{\underset{j=0}{\sum }}X_{ijk}q_{i}\leq Q,\;k=1,2,\ldots ,K,i\neq j$$

(22) $$t_{j}=\overset{N}{\underset{i=0}{\sum }}\overset{K}{\underset{k=1}{\sum }}X_{ijk}\left(t_{i}+\frac{d_{ij}}{v}+s_{i}\right),t_{0}=0,s_{0}=0,j=1,2,\ldots ,N$$

Equation (16) indicates that the goal of the model is to minimize the cost of unit satisfied customer. Equation (17) ensures that the vehicles used cannot exceed the number owned by the distribution center. Equation (18) imposes that all trucks must return to the depot when their distribution tasks are completed. Equations (19) and (20) represent that each customer is only visited once by one truck. Equation (21) shows that the total load on each route cannot exceed the rated load of the vehicle. Equation (22) indicates the time when the vehicle arrives at a customer point.

## 4. Algorithm Description

### 4.1. Algorithm Steps Design

The design of CEGA for an evolutionary cycle is as follows: Firstly, use the combination operator to make the population evolve with a certain evolutionary generations; secondly, update the population while preserving the best individual in history; finally, perform crossover and mutation operations on individuals according to crossover probability and mutation probability. Through completing the evolutionary cycles above until the termination condition is reached, CEGA finally finds an optimal solution.

(1) Coding

The code of this paper uses natural number coding.

(2) Producing feasible initial population at random

Generating N different chromosomes at random according to the initial population size N, this is the initial population$\;P_{0}$.

(3) Determining fitness function and fitness calculation

We take the reciprocal of the objective function value as the fitness value. The fitness function can be expressed as:

(23) $$F_{i}=10,000/r_{i}$$

where$\;F_{i\;}$represents the fitness value of individual i, and$\;r_{i\;}$represents the objective function value of individual i.

(4) Selection operation

We use the tournament selection [46] to conduct selection operation in this paper, which can ensure that the better individuals can be selected with higher probability, and the inferior individuals can be removed, thereby obtaining a quick convergence speed.

(5) Crossover operation

Considering the particularity of the coding of the VRP, the cycle crossover method was used in this paper.

(6) Mutation operation

Inversion mutation, insertion mutation, and interchange mutation strategies are all adopted in this paper.

(7) Generating a new generation population

A new population will be generated by Steps (4), (5), and (6).

(8) Terminating condition

The termination condition is whether the number of evolution cycles is greater than the maximum number of evolution cycles. If the condition is met, the loop will break; otherwise, the loop will continue.

(9) Decoding

Finally, we need to decode the optimal solution obtained to get the actual operational plan.

### 4.2. Parameter Setting for CEGA

The parameter setting for the CEGA has a considerable influence on the algorithm’s ability to solve the problem, and affecting the results of the model. A better solution can be obtained through the appropriate number of generations and mutation probability [47]. We refer to references [47,48,49], and the parameters are set as follows: The number of generations is 5000; the crossover probability is 0.4; the mutation probability is 0.05; the number of iterations of an evolution period is 10; and the initial population is 100.

## 5. Computational Experiments

Firstly, the CEGA proposed in this paper was tested in Section 5.1 by using the typical VRP test database. Secondly, the effectiveness of the VRP-CSC model was verified by an actual example, and the impact of carbon price on the model was further analyzed.

### 5.1. Algorithm Experiment

In this section, the benchmark database [50] is used to verify the effectiveness of CEGA. There are six test cases in the database (R1, C1, RC1, R2, C2, RC2), and this article randomly selects one problem from each type for algorithm testing. The algorithms tested in this section include standard genetic algorithms (GA) and cycle evolutional genetic algorithm (CEGA). The results are shown in Table 2.

From Table 2, six groups of experimental results were compared by the two algorithms. The results obtained by CEGA are 100% better than the results obtained by GA. Therefore, the CEGA has great advantages in obtaining high quality solutions.

### 5.2. Case Study

We consider the experiment based on a case study from a cold chain transport company [15]. The original authors studied the cold chain logistics path optimization problem under the carbon tax with the minimum total cost as the objective function in the case, but ignored the factor of customer satisfaction. Therefore, this paper considers cost, customer satisfaction, and carbon trading, and adopts the cost of unit satisfied customer as the objective function (instead of minimization of the total costs) to further study the cold chain logistics optimization problem. The location, demand, and desirable time windows of each customer are shown in Table 3. Relevant parameters of the refrigerated truck used are shown in Table 4. Other relevant parameters of the model are shown in Table 5. The average vehicle speed in the delivery process is 25 km/h, the rated load of the vehicle is 9 t. The carbon quota used in the experiment is the carbon emissions value of the initial solution in the algorithm, which is 100 kg. We use the carbon trading price of 1 CNY/kg by referring to reference [51].

### 5.3. Evaluation of the Model

**Figure 2:**
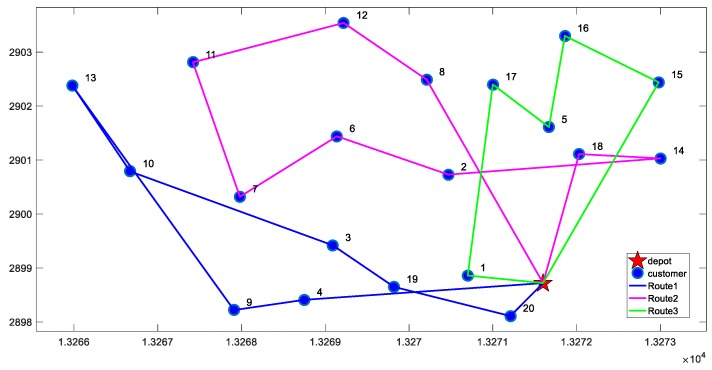
The optimal distribution paths of minimizing total cost.

**Figure 3:**
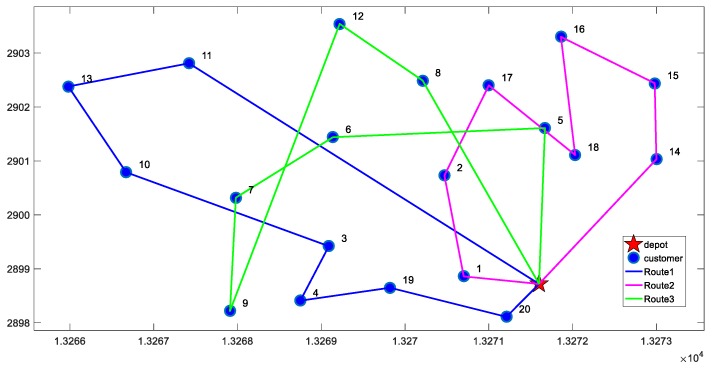
The optimal distribution paths of minimizing the cost of unit satisfied customer.

For the two cases of minimizing total cost and minimizing the cost of unit satisfied customer, we conducted 20 experiments separately. The results of each experiment are almost identical, and the optimal solutions under the two objective functions are shown in Figure 2 and Figure 3. The comparison of results is shown in Table 6.

From the results in Table 6, we can observe the following findings:

(1) According to the VRP-CSC model, the average customer satisfaction of results obtained is 70%, while it is only 40% when minimizing total cost is the objective function. Therefore, the VRP-CSC model can greatly improve customer satisfaction.
(2) In the meantime, the solution obtained by VRP-CSC model has higher carbon emissions. Therefore, we infer that there may be a trade-off between customer satisfaction and carbon emissions. This inference is confirmed by numerical analysis in Section 5.4.
(3) Through the comparison of r values, we found that the cost of unit satisfied customer obtained by VRP-CSC is less than the cost achieved by minimizing total cost. Adding 6.4% to total costs will achieve 70% level for average customer satisfaction. Thus, the solution obtained by VRP-CSC has higher cost-effectiveness.

In summary, this model achieves the expected results, indicating that the model is effective and scientific.

### 5.4. Analysis of Carbon Pricce

**Figure 4:**
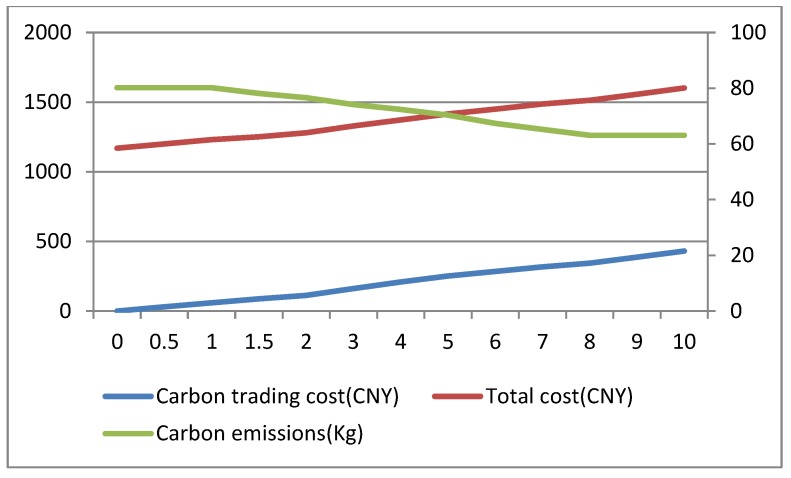
The changing trends of carbon emissions and total cost with the carbon price changes under insufficient carbon quota.

**Figure 5:**
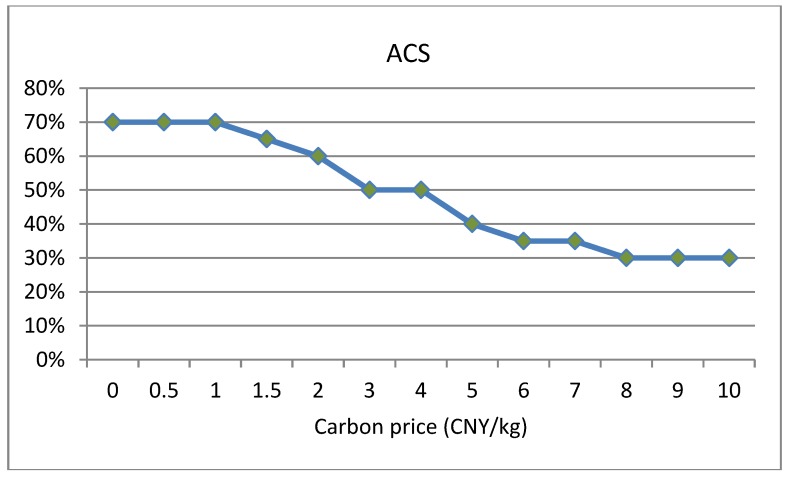
The changing trend of average customer satisfaction with the increase in carbon price under insufficient carbon quota.

**Figure 6:**
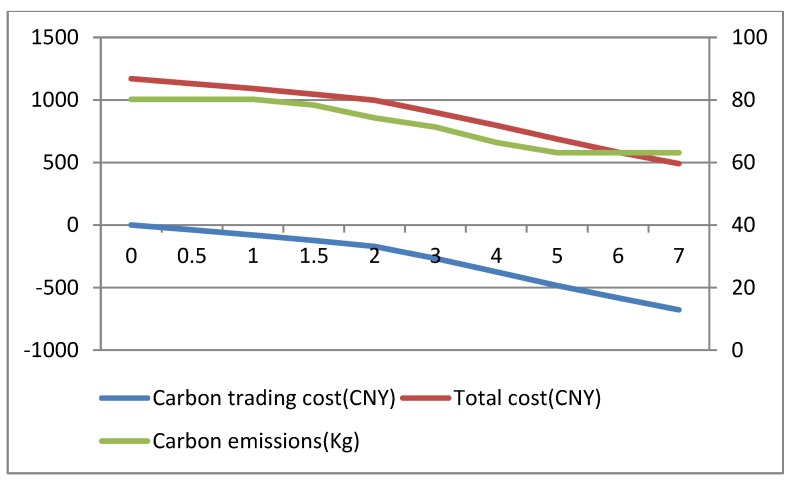
The changing trends of carbon emissions and total cost with the carbon price changes under sufficient carbon quota.

**Figure 7:**
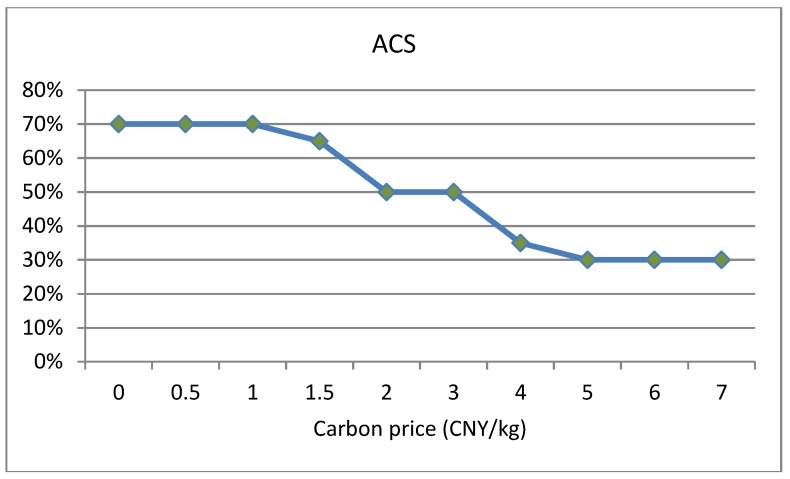
The changing trend of average customer satisfaction with the increase in carbon price under sufficient carbon quota.

Carbon price can serve as leverage for guiding resource allocation and optimization, which plays a determinative role in the carbon trading system [52,53]. This paper analyzes the impact of carbon price$\;C_{p\;}$(CNY/kg) on carbon emissions under two conditions: sufficient and insufficient carbon quota. In this section, in order to ensure insufficient or sufficient conditions for carbon quota under any solutions, we randomly set a smaller value (20 kg) as an insufficient carbon quota and a larger value (160 kg) as a sufficient carbon quota to conduct experiments respectively. The changing trends of carbon emissions, total costs and average customer satisfaction with the increase of carbon price under insufficient carbon quota are shown in Figure 4 and Figure 5. The changing trends of carbon emissions, total costs and average customer satisfaction with the increase of carbon price under sufficient carbon quota are shown in Figure 6 and Figure 7.

From results in Figure 3, Figure 4, Figure 5 and Figure 6, we can observe the following findings:

(1) When the carbon quota is insufficient, that is, the carbon emissions of a company are greater than the carbon quota, the total costs gradually increase with the increase in carbon price. When the carbon quota is sufficient, that is, the carbon emissions of a company are less than the carbon quota, the total costs gradually reduce with the increase in carbon price. Equation (15) shows that carbon trading costs are closely correlated with the difference between carbon emissions and carbon quota. If the carbon quota is insufficient, the difference is positive, resulting in carbon trading expenditures, and the total costs will gradually increase. On the contrary, the other scenario will generate carbon trading revenue, and the total costs will gradually decrease.
(2) From Figure 4, under the insufficient quota condition, we can see that when carbon price$\;C_{p\;}\in \left[0,\;1\right]$, carbon emissions remain unchanged; when the carbon price$\;C_{p\;}\in \left[1,\;8\right],$ there are continuous reductions in carbon emissions; when carbon price$\;C_{p\;}\in \left[8,+\infty \right],\;$carbon emissions basically remain unchanged. Similarly, from the Figure 6, under the sufficient quota condition, there are also three stages in the trend of carbon emissions. Therefore, whether the the carbon quota is sufficient or not, there will be an effective carbon price interval$\;\left[C_{p-lower,}\;C_{p-upper}\right]$ that reduces carbon emissions. As the result shows, cold chain logistics enterprises can reduce the total cost of distribution by optimizing the paths when the carbon price gradually increases in the effective interval. Objectively, there are also better environmental benefits.
(3) In the meantime, we can see that the overall average customer satisfaction is on a downward trend with the increase in carbon price within the effective carbon price interval, and changes in average customer satisfaction follow changes in carbon emissions. Hence, increases in carbon price not only reduce carbon emissions, but also reduce customer satisfaction to a certain extent. In addition, from the trend relationship between carbon emissions and average customer satisfaction, it can be seen that there is a certain trade-off between the two. Hence, the inference in Section 5.3 is confirmed. This is also consistent with the conclusion presented in reference [31]. As the results show, the government must properly control the carbon price. Low prices cannot achieve the effect of reducing emissions, and high prices will reduce customer satisfaction to a certain extent, thereby reducing the competitiveness of enterprises.

## 6. Discussion and Managerial Implications

For the vehicle routing optimization problem in cold chain logistics distribution, the VRP-CSC model is proposed in this paper to aim for optimization in terms of comprehensive consideration of cost, customer satisfaction, and carbon emissions. By taking the minimum cost of unit satisfied customer as the objective function, a distribution scheme that takes into account economic, customer experience, and environmental benefits can be obtained, thereby achieving a comprehensive optimization considering multiple factors. The main conclusions of this paper are summarized as follows:

1. The VRP-CSC model can simultaneously take into account cost, customer satisfaction, and carbon emissions factors, resulting in a highly cost-effective solution.
2. In the carbon trading mechanism, there is an effective carbon emissions reduction interval when carbon price$\;C_{p}\in \left[C_{p-lower,}\;C_{p-upper}\right]$.
3. There is a certain trade-off between customer satisfaction and carbon emissions.

For cold chain logistics enterprises, under fierce market competition and the demand for low-carbon economy, customer satisfaction and green logistics are key issues that have to be faced. This study points out that there is a trade-off relationship between customer satisfaction and carbon emissions. Therefore, it is a wise choice to seek a balance point between these two factors for the development of a company. This paper makes the following recommendations for this issue:

(1) If the customers have a strong dependence on the enterprise, the market competition is not very intense, and the cost for customers to change suppliers is relatively high, the enterprise should prefer the distribution plan with the lowest total cost.
(2) If the market competition is fierce, customers can choose more suppliers, and the cost for customers to change suppliers is relatively low, enterprises should pay attention to customer satisfaction, while considering carbon emissions.
(3) Enterprises can introduce low-cost, environmentally-friendly transportation equipment, and even adopt new energy transportation vehicles to reduce carbon emissions, which will save energy and reduce emissions without hurting customer satisfaction.
(4) From the perspective of long-term development, cold chain companies should develop joint distribution transportation modes, which can not only reduce emissions but also improve customer satisfaction [23,24].

For the government, the setting of carbon price should be scientific and reasonable. Neither too low nor too high carbon price can produce effective reduction of carbon emissions. Only in the effective range of carbon price can carbon price can play an effective role in carbon emission reduction. In addition, due to a trade-off between customer satisfaction and carbon emissions, high carbon prices will drive companies to take more low-carbon distribution routes, but at the same time, companies will also lose a certain degree of customer satisfaction, thereby decreasing competitiveness. Therefore, a reasonable carbon price standard is conducive to energy conservation and emissions reduction without affecting economic development of logistics firms.

## 7. Conclusions

As people pay more attention to the consumer experience and the arrival of a low-carbon economy, customer satisfaction and carbon emissions have become key issues for many industries. Cold chain logistics is a service-focused and high-carbon emissions industry; it is necessary to optimize the distribution network of cold chain logistics while taking into account customer satisfaction and environmental benefits. In this paper, based on the idea of cost–benefit, a comprehensive VRP-CSC model, with minimized cost of unit satisfied customer as the objective function, was designed to optimize cold chain distribution paths. An improved genetic algorithm, CEGA, is introduced to solve the model. Moreover, the numerical experiments are used to verify the effectiveness of the algorithm. Then, actual case data is used with the algorithm to carry out a computational experiment, and the experiment results show that a highly cost-effective solution can be obtained. Furthermore, with regards to the carbon trading mechanism, the impact of carbon price on total costs, carbon emissions, and average customer satisfaction has also been numerically analyzed. We also found that as carbon price increases, there are two opposite trends in total costs, depending on whether carbon quotas are sufficient; increasing carbon prices within a certain carbon price range can effectively reduce carbon emissions, but at the same time it will reduce average customer satisfaction to a certain extent, and there is a trade-off between carbon emissions and customer satisfaction. Finally, based on the conclusions above, some practical managerial implications for enterprises and government are presented.

Further research is required to consider more factors to evaluate customer satisfaction (e.g., the freshness, the loss rate, and so on), so that the model will be more adaptable to real-life scenarios.

## Figures and Tables

**Table 1:** Description of the symbols.

Symbols	Description
N	The number of customers (0, 1, 2,…, N; 0 is depot)
K	The number of trucks owned by the depot
Q	The rated load of the truck
$f_{k}$	The fixed cost of the truck
$d_{ij}$	The distance between customer *i* and *j*
$t_{i}$	The arrival time to customer *i*
p	The price of the unit weight goods
∂	The spoilage rate
$C_{e}$	The refrigeration costs which generate during transportation process of unit time
$C_{e}{}^{\prime }$	The refrigeration costs which generate during unloading process of unit time
$q_{i}$	The demand of customer *i*
$Q_{in}$	The weight of goods remaining on the vehicle when the refrigerated truck leaves customer *i*
∅	The coefficient value of CO_2_ emission
$C_{r}$	The unit fuel price
$C_{p}$	The unit carbon trading price
$Q_{q}$	The carbon quota which is allocated by government
$F_{c}$	The total fuel consumption in the entire distribution
ρ	The fuel consumption per unit distance when the vehicle is running
$\rho_{0}$	The fuel consumption per unit distance when the vehicle is empty
$\rho^{*}$	The fuel consumption per unit distance when the vehicle is fully loaded
$Q_{ij}$	The load of goods to be delivered when it travels between customer *i* and customer *j*
$\rho \left(Q_{ij}\right)$	The unit distance fuel consumption when the cargo weight carried by the vehicle is $Q_{ij}$
ACS	The average customer satisfaction
$Y_{0ik}$	0–1 variable, $Y_{0ik}=1$ if the vehicle k is used from depot to customer i, otherwise $Y_{0ik}=0$
$X_{ijk}$	0–1 variable, $X_{ijk}=1$ if the vehicle k visits customer j from customer i, otherwise $X_{ijk}=0$
$\theta_{i}$	0–1 variable, $\theta_{i}=1$ if the customer feels completely satisfied, otherwise $\theta_{i}=0$
$Z_{i}$	0–1 variable, $Z_{i}=1$ if the customer *i* is served, otherwise $Z_{i}=0$

**Table 2:** Test results of cycle evolutionary genetic algorithm (CEGA).

Problems	GA		CEGA	
	Number of Vehicles	Distance	Number of Vehicles	Distance
R1-01	19	1701.92	18	1647.32
C1-04	10	881.23	10	831.58
RC1-08	10	1396.51	10	1139.82
R2-02	6	1242.34	3	1191.70
C2-06	4	685.43	4	654.91
RC2-07	4	1259.31	3	1061.14

**Table 3:** Customer information.

Number	Coordinates (km)	Demand (t)	Desirable time	Service Time (min)
0	(13,271.60, 2896.72)	0	5:30–17:00	0
1	(13,270.70, 2898.86)	1.50	6:00–8:00	20
2	(13,270.47, 2900.73)	0.50	7:30–9:00	10
3	(13,269.09, 2899.42)	1.50	6:00–8:00	20
4	(13,268.75, 2898.41)	1.50	6:30–8:20	20
5	(13,271.67, 2901.61)	2.00	6:40–8:30	25
6	(13,269.14, 2901.44)	2.00	7:00–9:00	25
7	(13,267.98, 2900.32)	1.80	7:20–9:00	22
8	(13,270.21, 2902.49)	1.00	7:30–9:00	15
9	(13,267.91, 2898.22)	1.00	7:00–8:30	15
10	(13,266.67, 2900.79)	1.00	7:00–9:00	15
11	(13,267.42, 2902.81)	1.00	7:30–9:30	15
12	(13,269.22, 2903.54)	0.50	7:30–9:00	10
13	(13,265.98, 2902.38)	0.50	7:30–9:30	10
14	(13,273.00, 2901.03)	1.50	7:30–9:00	20
15	(13,272.98, 2902.44)	2.00	6:50–8:30	25
16	(13,271.86, 2903.30)	1.50	7:00–8:40	20
17	(13,271.00, 2902.40)	1.50	7:00–8:40	20
18	(13,272.03, 2901.11)	0.50	7:50–9:00	10
19	(13,269.82, 2898.65)	2.50	6:30–8:30	30
20	(13,271.21, 2898.11)	1.00	7:50–9:00	15

**Table 4:** Vehicle parameters.

Parameters	Parameter Values	Parameters	Parameter Values
Outline dimension	9990 × 2490 × 3850 mm	Container size	7400 × 2280 × 2400 mm
Total mass	16,000 kg	Rated load capacity	9000 kg
Engine type	B19 033	Fuel type	Diesel oil
No-load fuel consumption	16.5 L/100 km	Integrated fuel consumption	23.3 L/100 km

**Table 5:** Relevant parameters of the model.

Parameter	Value
P $\partial_{1},\partial_{2}$ $C_{e},C_{e}{}^{\prime }$	2000 CNY/t 0.002, 0.003 15 CNY/h, 20 CNY/h
$C_{r}$	7.25 CNY/L
∅	2.63 kg/L
$\rho_{0}$	0.165 L/km
$\rho^{*}$	0.377 L/km
$f_{k}$	200 CNY

**Table 6:** Comparison of results.

Objective Function	Minimize Total Cost	Minimize the Cost of Unit Satisfied Customer
Total cost	1080.25 CNY	1149.84 CNY
Carbon emissions	71.8 kg	80.2 kg
ACS	40%	70%
r	135.03 CNY	82.13 CNY

r is the value of objective function.
